# What is the effect of commercial food and non-alcoholic beverage marketing on the dietary intake of children and adolescents? Protocol for systematic review and meta-analysis with an equity lens

**DOI:** 10.1186/s13643-025-02978-x

**Published:** 2025-11-26

**Authors:** Qiuyu Julia Chen, Hadis Mozaffari, Jaithri Ananthapavan, Brendan T. Smith, Gavin W. K. Wong, Mahsa Jessri

**Affiliations:** 1 Food, Nutrition, and Health Program, Faculty of Land and Food Systems, The University of British Columbia, Vancouver, BC Canada; 2 Deakin Health Economics, School of Health and Social Development, Faculty of Health, Institute for Health Transformation, Deakin University, Geelong, VIC Australia; 3 Global Centre for Preventive Health and Nutrition, School of Health and Social Development, Faculty of Health, Institute for Health Transformation, Deakin University, Geelong, VIC Australia; 4 Public Health Ontario, 661 University Avenue, Suite 1701, Toronto, ON Canada; 5 Dalla Lana School of Public Health, University of Toronto, Toronto, ON Canada; 6 School for Population and Public Health, University of British Columbia, Vancouver, BC Canada; 7 Centre for Clinical Epidemiology and Evaluation, Vancouver Coastal Health Research Institute, Vancouver, BC Canada; 8 Centre for Health Services and Policy Research (CHSPR) and Health Services and Policy (HSP), Faculty of Medicine, The University of British Columbia, Vancouver, BC Canada

**Keywords:** Children, Food and beverage marketing, Advertising, Dietary intake, Socio-economic factors

## Abstract

**Background:**

Previous reviews have shown that food and beverage marketing increases dietary intake among children. However, no updated review has been conducted since the COVID-19 pandemic. Furthermore, limited evidence exists on how this effect varies by sociodemographic factors and marketing media. The proposed study aims to assess and quantify the effect of food and beverage marketing exposure on children’s dietary intake and to examine variations based on age, sex, ethnicity, socioeconomic position, and marketing medium.

**Methods:**

A systematic review of peer-reviewed primary studies will be conducted by including studies on dietary intake from two previous reviews conducted for the World Health Organization (WHO) from 1970 to March 2020, and by searching 19 databases from April 2020 to October 2024. The literature search will be supplemented with backward citation searching of retrieved reviews and included studies. The search terms will encompass three key concepts: food- and beverage-related marketing, dietary intake, and population. Eligible studies must assess the impact of advertising on dietary intake. Two independent reviewers will conduct literature review, data extraction, and quality assessment, with discrepancies resolved by consensus with a third reviewer. Data will be extracted into a standardized evidence table and, where appropriate, included in a meta-analysis to synthesize quantitative findings. Risk of bias will be assessed using the Risk of Bias 2 tool for randomized controlled trials and the Newcastle–Ottawa Scale for non-randomized studies. Publication bias will be examined using funnel plots and trim-and-fill methods, while heterogeneity will be assessed with the *I*^2^ statistic. The overall quality of evidence will be evaluated using the GRADE approach.

**Discussion:**

This review will be the first comprehensive assessment of the effect of food and beverage marketing exposure on children’s dietary intake, encompassing studies from 1970 to 2024. It will also assess the potential heterogeneity of effects by sociodemographic groups and marketing media. The findings will inform evidence-based policies aimed at reducing marketing-driven dietary risks among children. Limitations include reliance on previous WHO-commissioned reviews for studies published before April 2020, which may introduce selection bias, and a focus on acute exposure, potentially limiting applicability to real-world, long-term effects.

**Systematic review registration:**

PROSPERO CRD42025641870.

**Supplementary Information:**

The online version contains supplementary material available at 10.1186/s13643-025-02978-x.

## Background

Food and beverage marketing has become a pervasive driver of unhealthy dietary habits among children worldwide [1]. Research consistently shows that food and/or non-alcoholic beverage (hereafter referred to as "food") marketing targeted at children predominantly promotes foods high in fats, sugar, and salt (HFSS), which are typically ultra-processed and nutritionally poor compared to foods marketed to the general population [2–5]. HFSS food consumption is strongly linked to childhood obesity [6, 7], which increases the risk of chronic diseases like type 2 diabetes mellitus and cardiovascular conditions later in life [8, 9]. Therefore, international organizations, such as the World Health Organization (WHO), the United Nations Food and Agriculture Organization, and the Pan American Health Organization, have urged governments to implement policies and regulations that restrict children’s exposure to commercial marketing of HFSS foods to mitigate its adverse effects on dietary behaviors and long-term health outcomes [10, 11]. However, it is crucial to examine the complete evidence base to inform evidence-based policy development through a comprehensive and systematic review of the literature.

Evidence on the effects of food marketing on children’s dietary behaviors remains fragmented, and no systematic review and meta-analysis has comprehensively synthesized the relevant literature to provide a complete overview. For instance, two WHO-commissioned reviews by Cairns et al. [12] and Boyland et al. [13] have found that increased dietary intake is one of the major outcomes of food marketing exposure in children and adolescents. However, while the 2022 review was intended as an update to the 2009 report, it did not incorporate studies identified in the earlier review. This omission may limit a full understanding of the scope and consistency of findings over time. Additionally, the 2022 review included evidence from January 2009 to March 2020. Hence, there is over 4 years of new literature to be evaluated which include studies conducted during and after the COVID-19 pandemic, a period that significantly influenced children’s exposure to HFSS food marketing, particularly through digital media [14]. Therefore, a systematic review that synthesizes all relevant studies from previous reviews and incorporates updated evidence to compare the effects of food marketing exposure before 2009, between 2009 and 2020, and after 2020 would strengthen the evidence base and offer a clearer, more comprehensive understanding of the impact of marketing on children’s dietary intake and identify potential changes over time.

Furthermore, existing reviews have overlooked the role of sociodemographic factors in shaping marketing effects, despite these being important factors likely to modify the relationship between advertising and dietary intake [15]. While this omission does not necessarily suggest selective reporting bias—since the lack of segregated data may simply preclude such analyses—it highlights a significant gap that could impact the validity and generalizability of the findings. By combining studies included in previous reviews with newly eligible studies published after 2020, our review will attempt to generate sufficient data to enable subgroup analyses.

The effect of food marketing on children’s dietary intake is likely to be modified by age, sex, and socioeconomic position (SEP) due to key biological, cognitive, and environmental differences among these groups [16]. Research shows that younger children are more susceptible to marketing due to their limited cognitive abilities and inability to critically evaluate persuasive messages, whereas adolescents may have greater media literacy but are exposed to more targeted and personalized digital marketing [17–25]. Sex differences in responses to food marketing may arise from varying preferences, behaviors, and social norms around food consumption, as well as differences in susceptibility to marketing messages. For instance, evidence suggests that boys may respond more to promotions of energy-dense foods, while girls may be more influenced by health-related marketing [26–28]. Additionally, previous studies show that children from lower-SEP households are more likely to be exposed to marketing for unhealthy foods due to affordability, higher exposure to television advertising, and limited access to healthier alternatives [16, 29–31]. Identifying potential moderators is crucial for recognizing vulnerable subgroups and designing targeted interventions to mitigate the potential inequitable effects of marketing restrictions on children’s dietary intake.

Finally, the marketing medium might serve as another moderator of the effect of food marketing, influencing outcomes through variations in message delivery, engagement, and exposure frequency. For instance, digital media often involves personalized and interactive content, which may have a stronger impact compared to traditional media, such as television or print advertisements [32].

Therefore, the systematic review and meta-analysis outlined in this protocol aims to (a) quantify the effect of food marketing exposure on children’s dietary intake, with particular attention to changes over time; (b) examine variations in this effect across sociodemographic factors, including age, sex, ethnicity, and SEP; and (c) evaluate differences in the effect based on the marketing medium used.

## Methods/design

### Study design

This protocol will follow the Cochrane standard for systematic reviews [33] and Preferred Reporting Items for Systematic Reviews and Meta-Analyses (PRISMA) guideline [34]. The presentation of the final results will adhere to the PRISMA 2020 Checklist. This protocol followed the PRISMA-P 2015 Checklist (Supplement 1). The protocol was pre-registered with PROSPERO in February 2025 (CRD42025641870, available from https://www.crd.york.ac.uk/prospero/display_record.php?ID=CRD42025641870).

### Eligibility criteria

To be eligible for this systematic review, the articles must be empirical primary studies. Both experimental and observational studies will be included. The inclusion criteria are structured according to the PICO framework (Tables 1 and 2). The population criteria include children and adolescents (aged between 0 and 19 years). The intervention criteria encompass any form of food marketing, following the WHO guideline for policies to protect children from the harmful impact of food marketing; marketing is defined as “*any form of commercial communication, message or action that acts to advertise or otherwise promote a product or service, or its related brand, and is designed to increase, or has the effect of increasing, the recognition, appeal and/or consumption of products or services*” [35]. Mediums used for food marketing can include television programs, digital media (e.g., internet websites, video games, “advergames,” influencer marketing, user-generated content, and mobile apps), printed media (e.g., magazines and newspapers), product packaging, product placements at the retail stores, promotions in and around the schools, and other outdoor advertising (e.g., posters and moving vehicles) [12, 25, 36–39].

**Table 1:** PICO framework guiding study search strategy

Population (P)	Children aged 0–19 years
Intervention (I)	Exposure to commercial food or non-alcoholic beverage marketing (not including food supplements, vitamins, or infant formula)
Comparator (C)	No or less exposure to marketing for food and non-alcoholic beverages
Outcomes (O)	Food or non-alcoholic beverage intake, measured as energy intake, the quantity of item consumed, or frequency of consumption

**Table 2:** Inclusion and exclusion criteria

	**Inclusion criteria**	**Exclusion criteria**
Study participants	• Human studies • Children and adolescents (aged 0 to 19 years)	• Animal studies • Only includes adults (age 20 years and older) • Ages of participants are NOT reported
Intervention/exposure	• The intervention criteria encompass any form of food or non-alcoholic beverage marketing that meets WHO definition for marketing • WHO definition for marketing “any form of commercial communication, message or action that acts to advertise or otherwise promote a product or service, or its related brand, and is designed to increase, or has the effect of increasing, the recognition, appeal and/or consumption of products or services” [35] • The medium used for child-targeting food marketing can include television programs, digital media (e.g., internet websites, video games, “advergames,” influencer marketing, user-generated content, and mobile apps), printed media (e.g., magazines and newspapers), product packaging, product placements at the retail store, and promotions in and around the school	• Studies assessing the effect of advertising for infant formula or marketing strategies outside of the WHO’s definition will be excluded • Studies assessing the effect of television viewing only will be excluded
Comparator/context	No or less exposure to marketing for food and non-alcoholic beverages	
Outcome	The outcomes considered are food or non-alcoholic beverage intake, measured either as energy intake, the quantity of items consumed, or frequency of consumption	Other outcomes such as food choice, food preference, and purchasing behavior (by or on behalf of children) will be excluded
Study characteristics	Primary experimental studies of quantitative or mixed-method design (including RCTs, pre-post designs, quasi experimental studies) and observational studies (cross-sectional or longitudinal)	• Materials containing subjective or opinion-based information, such as editorials, commentaries, letters, or blogs, will be excluded • Reviews of studies (scoping, narrative or systematic) • Purely qualitative studies (focus groups, interviews, case studies) • Studies exclusively assessing marketing prevalence (exposure) and/or nature (power) • Noncomparative studies
Language study is reported in	No language restrictions	

The outcomes considered are food intake, measured either as energy intake or the quantity/frequency of items consumed. Studies assessing the effect of advertising for infant formula or marketing strategies outside of the WHO’s definition will be excluded. Additionally, other outcomes such as food choice, food preference, and purchasing behavior (by or on behalf of children) will be excluded because the focus of this review is specifically on the direct measurement of food intake. Materials containing subjective or opinion-based information, such as editorials, commentaries, letters, or blogs, will also be excluded due to the uncertainty of their peer review status. No language restrictions will be applied, as the databases typically translate titles and abstracts into English. Included non-English articles that are not in Persian or Chinese, which are the languages spoken by the research team, will be translated into English using Google Translate (https://translate.google.ca) for full-text review.

The search will be restricted to studies published from April 2020 onward, as the previous two WHO reviews included evidence from 1970 to March 2020 [12, 13]. Data will be extracted from eligible articles, combined with those from the WHO reviews, which measured the impact of marketing exposure on dietary intake in children, for quality assessment and analyses. Furthermore, the quality of the two WHO reviews will be cross-referenced with that of related reviews published after March 2020, identified through the search. Any eligible articles identified from related reviews will also be included for quality assessment and analyses.

### Information sources

Various databases were utilized to find eligible articles: *MEDLINE*, *CINAHL*, *Web of Science*, *Embase*, *ERIC*, *the Cochrane Library (CDSR*, *CENTRAL)*, *Business Source Ultimate*, *Communication & Mass Media Complete*, *Database of Promoting Health Effectiveness Reviews (DoPHER)*, *EconLit*, *Emerald*, *Global Index Medicus*, *Healthevidence.org*, *IRIS (Institutional Repository for Information Sharing)*, *JSTOR*, *KOREAMED*, *Index to Legal Periodicals & Books Full Text (H.W. Wilson)*, *The Campbell Library, and TRIP (Turning Research Into Practice)*. Targeted searches with citation chaining, both forward and backward, will also be conducted to supplement the term-based searching [40]. To ensure that no relevant publications are missed, backward citation chaining will be performed by reviewing articles in the bibliographic lists of related reviews and all included full-text articles identified through Scopus [41]. Forward citation chaining of all included full-text articles will be undertaken with citationchaser [42].

### Search strategy

The search strategy was designed to capture generic terms for children and adolescents (population), food and beverage marketing (intervention), and dietary intake (outcome) in both observational and experimental studies. The population and intervention components of the search strategy were adopted from the systematic reviews conducted by Boyland et al. [13] and Cairns et al. [12]. The outcome component was developed by the review authors with help from an information specialist. We have also consulted a health science librarian specializing in human nutrition (KM) at the University of British Columbia to further review and test the sensitivity of the search strategies and techniques. Specifically, hand selection of 30 articles from Boyland et al. [13] on dietary intakes (Supplement 2) was used as benchmarking articles to evaluate the sensitivity [43] of the outcome component of the search strings with Medline. All benchmarking articles indexed in Medline were successfully captured and showed a search string sensitivity of 100% (Supplement 3). The search strategies used for all databases are presented (Supplement 4). The primary reviewer (QC) conducted the initial systematic search for relevant literature in October 2024, and the accuracy of all searches was peer-reviewed by two authors (HM and GW).

### Study records

#### Data management

Covidence, a systematic review software with Data Extraction 2.0, is used to organize, review, and extract data from the database results [44]. The included articles will be exported to Zotero for reference management and citation handling [45]. The data extracted from included articles and used in analyses, the annotated analytical code in R, and a detailed data dictionary will be deposited in the University of British Columbia Dataverse Collection on Borealis to ensure transparency, enable data reuse, and support replication of our findings [46].

#### Selection process

**Fig. 1:**
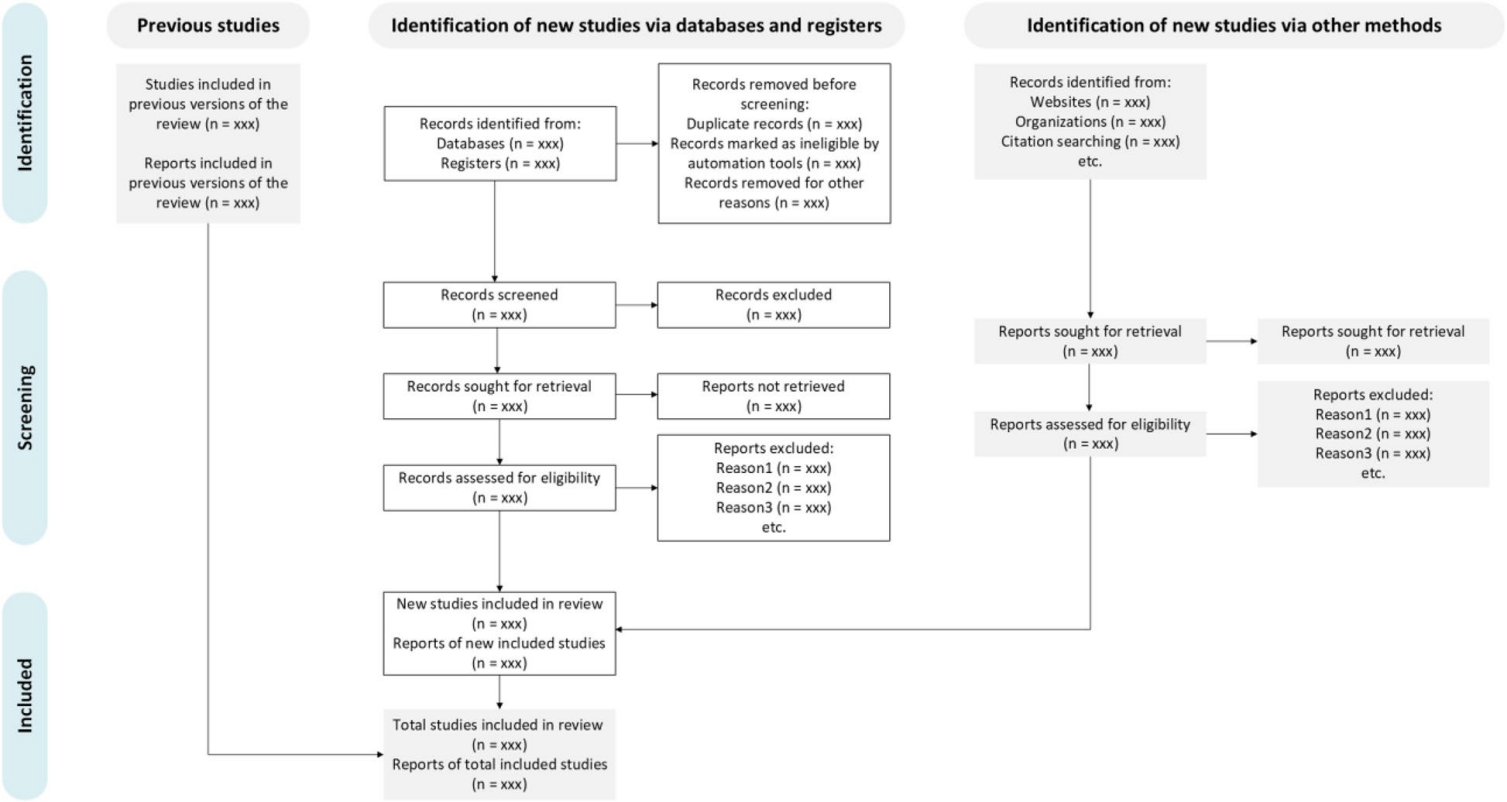
PRISMA flow diagram

We aimed to update the previous reviews conducted for the WHO [12, 13] which searched up to April 2020. We conducted an electronic search for recent studies from that date onward. All identified records from databases were imported into Covidence, which automatically detected and eliminated duplicate results. Following this, two reviewers will independently screen the results in a two-stage process. Initially, titles and abstracts will be independently reviewed by each reviewer, with eligibility categorized as “yes” or “no” based on the inclusion criteria. All titles labeled as “yes” will proceed to full-text screening for further eligibility assessment. Reasons for excluding articles at the full-text stage will be documented and presented in a PRISMA flow diagram (Fig. 1) [47].

The double-screening approach aims to ensure consistency in the screening process and follow the Cochrane standard. Any disagreements or discrepancies will be resolved through consensus and consulting a third reviewer as necessary. If conflicts remain unresolved, the senior author (MJ)—an epidemiology expert with extensive experience in nutrition research—will serve as an arbitrator and make the final decision. All decisions made to resolve discrepancies, along with their rationale, will be documented.

At the start of the title and abstract screening, we conducted a pilot test using a random sample of 80 articles. Two reviewers independently screened these references using the inclusion/exclusion criteria. Discrepancies were discussed until consensus was reached, and refinements were made to the criteria wording for clarity. Although none of these articles met the inclusion criteria, reviewer agreement reached over 90%, indicating a reliable screening approach. Adjustments were also made to exclude studies that have commercial food or non-alcoholic beverage marketing as part of their intervention but whose effect is not measured separately from the rest of intervention component, such as nutrition education. Additionally, the definition of outcome was refined to be food and non-alcoholic beverages consumed by participants. Studies measuring intention to consume will be excluded.

#### Data extraction process

In alignment with PRISMA recommendations to implement strategies to reduce data extraction error, data from a single study will be extracted independently by two reviewers using Covidence [44]. In addition, Covidence will allow a third reviewer to compare data extraction and bring discrepancies to the team to discuss and reach consensus [44]. A detailed description of all extracted variables, developed through piloting with independent reviewers, is implemented in the Covidence data extraction form to ensure the validity and consistency of the extracted data. It follows structured Covidence sections, including study identification, study methods, study population, study interventions, and study outcomes (Table 3). If needed, study authors will be contacted to provide data. PlotDigitizer will be used to extract numerical data from figures [48]. To minimize random error, two independent reviewers will extract data from the same study, and consensus will be reached through team discussion. The extracted data in the Covidence consensus form will then be further validated by the primary reviewer (QC).

**Table 3:** Data extraction form, adopted from Covidence. Customized for the systematic review protocol: “What is the effect of commercial food and non-alcoholic beverage marketing on the dietary intake of children and adolescents?” (PROSPERO CRD42025641870) based on data extraction form by Covidence: A practical guide: Data Extraction for Intervention Systematic Reviews. 2024

Study identification		
Study ID	Unique number assigned by Covidence and name that identifies the study	Number and last name of the first author_publication year
Contact author details	Contact author's name, institution, email	Narrative description
Country study was conducted in	Where the study was conducted	Narrative description
Setting	Note the type of place or environment where the study was conducted in, such as hospital, outpatient clinic, workplace, or school	Narrative description
Sponsorship source	How this study was funded	Narrative description
**Study methods**		
Study aim	The purpose of the study	Narrative description
Study design	Type of the study design (e.g., cross-sectional, longitudinal, and randomized control trail with method of randomisation)	Narrative description
**Study population**		
Inclusion criteria	Criteria used to define who was eligible for the study	Narrative description
Exclusion criteria	Criteria used to define who was not eligible for the study	Narrative description
Total sample size	How many participants were included in the study	Number
Withdrawal from study	Number, reason, and timing of withdrawals	Narrative description
*Baseline characteristics (for both intervention and control group)*		
Mean age	Mean age (years) ± SD	Number
Sex	Number of participants for each sex	Male: number Female: number
Ethnicity	Number of participants from each ethnicity group	Ethnicity group: number
Socio-economic position (SEP)	Number of participants from each SEP (e.g., income level, urban vs. rural, highest education attained in the household)	SEP: number
**Study interventions**		
Intervention definition	Definition of the intervention as described in the study	Narrative description
Control definition	Definition of the control as described in the study	Narrative description
Number allocated to intervention/comparison group	Number of participants who were allocated or randomized to the intervention and comparison group	Number
Duration of intervention	Duration of marketing exposure for the intervention and comparison groups	Number/frequency (narrative description)
**Study outcomes**		
Outcome definition	Name of the outcome measured (e.g., a single food item, a group of healthy foods, unhealthy foods, or both)	Narrative description
Type of outcome and default measures	Whether the outcome is a continuous or dichotomous variable and how it is reported for each group (e.g., mean difference, standard deviation, 95% confidence interval, events, odds ratio, standard error)	Narrative description
Timepoint	Timepoint(s) of intervention effects measured	Baseline, at measured point during treatment, end of treatment, at follow-up after treatment
Scale	The name of the scale used to measure the outcome	Narrative description
Range	The upper and lower limits of the scale	Number
Unit of measurement	Such as kilocalorie, kilojoule, gram	Narrative description

### Quality assessment

We will use the Risk of Bias 2 (RoB 2) tool [49] to assess bias in randomized controlled trials (RCTs) and adopt the Newcastle–Ottawa Scale (NOS) [50] to evaluate the quality of non-randomized studies. The RoB 2 tool [49] will evaluate the quality of evidence based on 22 questions across five domains: the randomization process (3 questions), adherence to or deviations from the intended intervention (7 questions), objective measurement of outcomes (4 questions), missing outcome data (5 questions), and selective reporting of results (3 questions). Each item within a domain will have five response categories: yes, probably yes, no, probably no, and not indicated. Based on these assessments, studies will be classified into three categories: low risk of bias (high quality), some concern for bias (medium quality), and high risk of bias (low quality).

For non-randomized studies, we initially piloted three cross-sectional studies using the ROBINS-E tool [51] but ultimately opted to modify an existing NOS version developed for nutrition-related cross-sectional studies [52]. While ROBINS-E offers a robust conceptual framework for evaluating non-randomized exposures, we found it challenging to apply consistently—particularly when studies lacked clear temporality between exposure and outcome or did not align with a well-specified causal model, which is often the case in cross-sectional nutrition research. To address these challenges, we developed a customized NOS for cross-sectional studies focused on food marketing and dietary intake in children and adolescents (Supplement 5), based on the NOS criteria developed for cross-sectional studies on the food environment by Blanchard et al. [52]. The tool includes structured criteria across four domains—selection, comparability, exposure assessment, and outcome assessment—with explicit rules for assigning high-, moderate-, or low-quality ratings. Special emphasis is placed on the accuracy of exposure measurement and the appropriateness of statistical analysis, both of which are critical to evaluating the validity of findings. Our adaptation draws on relevant elements of ROBINS-E [51] and previous NOS modifications [52]. As for cohort studies, we will use a version of NOS modified for longitudinal nutrition studies with a scoring system (Supplement 6) [53]. These tools were pilot tested by two independent reviewers to ensure clarity and consistency before full application.

Moreover, we will use the Instrument to Assess the Credibility of Effect Modification Analyses (ICEMAN) [54] to evaluate the credibility of subgroup findings from randomized controlled trails in this review. The ICEMAN tool [54] is a validated instrument designed to systematically evaluate whether observed subgroup differences are likely to reflect true effect modification or are more plausibly explained by bias, chance, or other confounding factors. The tool considers several key criteria, including whether subgroup hypotheses were pre-specified, whether the subgroup variables were measured at baseline, the consistency of effect modification across studies, the statistical strength of interaction tests, and the biological plausibility of the observed differences. Subgroup analyses—such as those based on age, sex, socioeconomic position, or marketing medium—will be evaluated using the ICEMAN checklist.

Furthermore, we will apply the Grading of Recommendations, Assessment, Development, and Evaluation (GRADE) approach [55] to assess the overall certainty of the body of evidence across studies. GRADE evaluates the certainty of evidence based on five key domains: (1) risk of bias, (2) inconsistency of study results, (3) indirectness of evidence, (4) imprecision of effect estimates, and (5) publication bias. For each outcome, the body of evidence will be assessed separately for randomized and non-randomized studies. In line with GRADE guidance, evidence from randomized controlled trials will initially be rated as “high certainty,” while evidence from non-randomized studies will start as “low certainty.” These ratings may then be downgraded or upgraded depending on the evaluation of the five domains. For instance, non-randomized studies with a low risk of bias, a large effect size, and evidence of a dose–response relationship may be upgraded. Conversely, randomized trials with methodological limitations or high inconsistency may be downgraded. The final certainty of the evidence will be classified as high, moderate, low, or very low. High certainty indicates that further research is very unlikely to change confidence in the estimate of effect, while very low certainty suggests that the true effect is likely to be substantially different from the estimate.

### Outcomes

The outcomes considered in the studies encompass both food and non-alcoholic beverage intake. These outcomes are evaluated through two primary measures: the total energy intake and the quantity/frequency of items consumed. Total energy intake refers to overall caloric consumption, which is typically quantified in kilocalories per day. On the other hand, the quantity of items consumed can be assessed in several ways, including the number or frequency of servings or portions, the specific sizes of these portions, or the total weight or volume consumed.

### Data synthesis

The systematic review and meta-analysis process will be presented using PRISMA’s four-phase flow diagram [47]. The included research will be synthesized through meta-analysis with *metafor* package in R whenever possible and appropriate [56]. Study outcomes will be combined via standardized mean difference or odds ratio in the meta-analysis when studies are sufficiently homogeneous. Narrative synthesis will be conducted when meta-analysis is not feasible. For meta-analyses, random-effects restricted maximum likelihood estimator analysis will be conducted using the package in R. Any influential cases with a difference in beta score greater than 1 will be examined. The *I*^2^ statistic will be used to evaluate heterogeneity. A one-way sensitivity (leave-one-out) analyses will be conducted by excluding one study at a time to ensure no individual study drives the pooled results. Trim-and-fill analyses will be conducted to estimate the outcomes of missing studies and adjust the meta-analysis accordingly to account for publication bias when appropriate. Graphical displays of heterogeneity will include funnel plots, which can indicate publication bias if small studies with non-significant effects are missing from the published literature, and contour-enhanced funnel plots, which can distinguish between publication bias and other possible causes of asymmetry.

Subgroup analysis will also be performed by sociodemographic variables, including sex, age group (children vs. adolescents), and SEP, and marketing channel (e.g., television vs. digital vs. packaging) whenever appropriate. If sufficient data are available, we will also conduct a subgroup analysis comparing the effects of marketing for HFSS foods versus non-HFSS foods. If sufficient studies are available (≥10 per moderator), we will additionally perform meta-regression to assess the impact of continuous study-level moderators (e.g., duration of exposure). Additionally, we will conduct meta-analyses stratified by study design or explore study design as a covariate in meta-regression models to understand its influence on effect sizes. Finally, *p*-curve analyses will be conducted using the dmetar function in R with the *metafor* package to examine the evidential value [56].

## Discussion

This systematic review and meta-analysis will provide a timely update to the previous WHO reviews, which included studies published between 1970 and 2020. It will be the first to comprehensively synthesize the effects of food and beverage marketing on children’s dietary intake, using evidence spanning 1970 to 2024. Therefore, it will be able to provide a comparison of the effects of marketing exposure before and after the COVID-19 pandemic. The results will contribute to the growing evidence of the effects of marketing exposure and inform the development of population-level regulations. Additionally, the up-to-date and quantified intervention effects can be used as inputs to model the cost-effectiveness of food marketing regulations, particularly in terms of diet-related health outcomes, mitigating health inequities and healthcare cost savings [57].

A key strength of this study is its quantification of subgroup effects by age, sex, and SEP, which were not addressed in previous reviews [1, 13, 16, 16, 58–60]. These demographic factors are crucial for understanding how advertising influences dietary intake. For instance, a prior study demonstrated that children from socioeconomically and ethnically disadvantaged households are disproportionately exposed to food marketing and may, in turn, have higher consumption of unhealthy food [16]. Also, a Canadian study further identified significant sex differences in children’s exposure to food advertising on television [61]. For example, male children in Vancouver and Montréal were exposed to 24.7% and 24.0% more unhealthy food advertisements per person per year, respectively, compared to females in 2019 [61]. It might be important for stakeholders to take sex into account in the development, implementation, and monitoring of food advertising restrictions. For countries and regions with food marketing regulations, the definition of age ranges varies from under 12 years old to under 18 years old [35]. Although the inclusion of adolescents in such regulations may be supported by consumers and health stakeholders as a means to reduce teens’ marketing exposure and promote dietary health, it may be opposed by industry stakeholders due to concerns that it could impact their ability to reach adult audiences [62]. Therefore, given the ongoing debate among stakeholders regarding age definitions in food marketing regulation policies, understanding how marketing exposure differentially impacts dietary behavior in young children and adolescents is crucial for evidence-based policymaking [39].

Another strength lies in the planned subgroup analysis by marketing medium, especially because most of the existing reviews have evaluated the impact of a specific type of marketing medium [59, 60, 63]. For example, the study by Russell et al. [59] focused only on the impact of screen advertising but did not consider other forms of marketing, such as in-store promotions, product packaging, and digital marketing campaigns. Given that different government regulations target various marketing platforms, understanding the specific impact of each is critical for shaping effective policies. For example, Canada has proposed restrictions on television and digital media marketing, while Chile enforces broader regulations across television, websites, schools, and packaging [64]. In contrast, the UK has restricted only television advertising and recently announced to extend this regulation to online marketing communications, despite being one of the earliest countries to regulate food marketing [65]. Our study will explore these variations, offering insights into how marketing across different media may differentially affect children’s dietary intake, providing evidence to inform regulatory strategies.

Finally, this systematic review and meta-analysis will be the first comprehensive assessment to quantify the effect of food marketing exposure on children’s dietary intake, encompassing studies published between 1970 and 2024. Additionally, previous reviews often conducted narrower database searches [66], frequently focused on systematic reviews without meta-analyses [1], emphasized unhealthy food and beverage marketing exclusively [60], or assessed a limited range of marketing media [59, 63]. To build on this foundation, our study aims to incorporate a broader set of studies to enable subgroup analyses by sex, age, and SEP. We will also expand the scope to include a wider range of marketing media and both healthy and unhealthy foods, while employing more rigorous search and analysis methods to provide a comprehensive assessment. Furthermore, previous reviews have typically used either GRADE or the RoB tool [1, 16], while our review integrates both to provide a more thorough and comprehensive evaluation. Although a high risk of bias identified by the RoB tool can lead to downgrading the certainty of evidence in GRADE, the two tools serve distinct purposes [67]. The RoB tool focuses on evaluating the risk of bias in individual studies (internal validity), whereas GRADE assesses confidence in the overall conclusions drawn from the entire body of evidence. Even when all studies demonstrate a low risk of bias, factors beyond the scope of the RoB tool—such as inconsistency, indirectness, and imprecision—may still affect the certainty of the evidence in GRADE [55]. Additionally, incorporating ICEMAN will strengthen the rigor of our subgroup analyses and enhance the reliability of our equity-focused conclusions.

It is also important to acknowledge certain limitations in our updated review. First, we will exclude studies published before April 2020 and instead rely on two previous reviews conducted for the WHO [12, 13] as a primary source. As a result, any potential selection bias inherent in previous work may also affect the current review. For example, we have found that two studies [29, 68] identified by the systematic review conducted by Backholer [16] but not by Boyland [13]. Therefore, we propose conducting citation searches for any relevant reviews identified through databases and registers. Any new study identified from these reviews, regardless of their publication date, will be included in our review to supplement and cross-reference the WHO reviews. Second, this review will only include the studies that assess responses to acute food marketing exposure, which can limit the generalizability of our findings to real-world settings. Third, our focus on studies that specifically assess the effect of marketing on children’s dietary intake may limit the applicability of our findings to other related areas, such as children’s broader food-related behaviors or long-term health outcomes.

## Supplementary Information

Supplementary Material 1.

## Data Availability

Data sharing is not applicable to this protocol, as no data was generated or analyzed. The findings from this systematic review will be shared through scientific conference presentations and a peer-reviewed publication. Any necessary amendments to the current protocol will be documented in the final publication, including the date of modification, description, and rationale. All data will be accessible in the published review.

## Abbreviations

GRADE: Grading of Recommendations Assessment, Development, and Evaluation
HFSS: High in fats, sugar, and salt
ICEMAN: Instrument to assess the Credibility of Effect Modification Analyses
NOS: Newcastle-Ottawa Scale
PRISMA: Preferred Reporting Items for Systematic Reviews and Meta-analyses
RCTs: Randomized controlled trials
RoB 2: Risk of Bias 2
ROBINS-E: Risk Of Bias In Non-randomized Studies—of Exposures
SEP: Socioeconomic position
WHO: World Health Organization
