# Supplementary material for: What is the effect of commercial food and non-alcoholic beverage marketing on the dietary intake of children and adolescents? Protocol for systematic review and meta-analysis with an equity lens
Source: Syst Rev. 2025 Nov 26;14:238. doi: 10.1186/s13643-025-02978-x (PMC12659392; doi:10.1186/s13643-025-02978-x)
Supplement: Supplementary file 1 — Supplementary Material 1. [file 13643_2025_2978_MOESM1_ESM.docx]

**Supplement 1. PRISMA-P 2015 Checklist**

# **This checklist has been adapted for use with systematic review protocol submissions to BioMed Central journals from Table 3 in Moher D et al**:**** Preferred reporting items for systematic review and meta-analysis protocols (PRISMA-P) 2015 statement. *Systematic Reviews* 2015 ****4****:1

# An Editorial from the Editors-in-Chief of *Systematic Reviews* details why this checklist was adapted - **Moher D, Stewart L & Shekelle P**:**** Implementing PRISMA-P: recommendations for prospective authors. *Systematic Reviews* 2016 ****5****:15

| **Section/topic** | **#** | **Checklist item** | **Information reported** | | **Line number(s)** |
| --- | --- | --- | --- | --- | --- |
|  |  |  | **Yes** | **No** |  |
| **ADMINISTRATIVE INFORMATION** | | | | | |
| **Title** | | | | | |
| Identification | 1a | Identify the report as a protocol of a systematic review |  |  | P. 1 |
| Update | 1b | If the protocol is for an update of a previous systematic review, identify as such |  |  |  |
| **Registration** | 2 | If registered, provide the name of the registry (e.g., PROSPERO) and registration number in the Abstract |  |  | Line 66 |
| **Authors** | | | | | |
| Contact | 3a | Provide name, institutional affiliation, and e-mail address of all protocol authors; provide physical mailing address of corresponding author |  |  | Line 1-18 |
| Contributions | 3b | Describe contributions of protocol authors and identify the guarantor of the review |  |  | Line 515-519 |
| **Amendments** | 4 | If the protocol represents an amendment of a previously completed or published protocol, identify as such and list changes; otherwise, state plan for documenting important protocol amendments |  |  |  |
| **Support** | | | | | |
| Sources | 5a | Indicate sources of financial or other support for the review |  |  | Line 514 |
| Sponsor | 5b | Provide name for the review funder and/or sponsor |  |  |  |
| Role of sponsor/funder | 5c | Describe roles of funder(s), sponsor(s), and/or institution(s), if any, in developing the protocol |  |  |  |
| **INTRODUCTION** | | | | | |
| **Rationale** | 6 | Describe the rationale for the review in the context of what is already known |  |  | Line 80-135 |
| **Objectives** | 7 | Provide an explicit statement of the question(s) the review will address with reference to participants, interventions, comparators, and outcomes (PICO) |  |  | Line 136-140 |
| **METHODS** | | | | | |
| **Eligibility criteria** | 8 | Specify the study characteristics (e.g., PICO, study design, setting, time frame) and report characteristics (e.g., years considered, language, publication status) to be used as criteria for eligibility for the review |  |  | Line 149-183 |
| **Information sources** | 9 | Describe all intended information sources (e.g., electronic databases, contact with study authors, trial registers, or other grey literature sources) with planned dates of coverage |  |  | Line 184-201 |
| **Search strategy** | 10 | Present draft of search strategy to be used for at least one electronic database, including planned limits, such that it could be repeated |  |  | Line 203-216 |
| ***STUDY RECORDS*** | | | | | |
| Data management | 11a | Describe the mechanism(s) that will be used to manage records and data throughout the review |  |  | Line 218-232 |
| Selection process | 11b | State the process that will be used for selecting studies (e.g., two independent reviewers) through each phase of the review (i.e., screening, eligibility, and inclusion in meta-analysis) |  |  | Line 233-258 |
| Data collection process | 11c | Describe planned method of extracting data from reports (e.g., piloting forms, done independently, in duplicate), any processes for obtaining and confirming data from investigators |  |  | Line 259-272 |
| **Data items** | 12 | List and define all variables for which data will be sought (e.g., PICO items, funding sources), any pre-planned data assumptions and simplifications |  |  | Line 263-271, Table 1, 3 |
| **Outcomes and prioritization** | 13 | List and define all outcomes for which data will be sought, including prioritization of main and additional outcomes, with rationale |  |  | Line 165-176, Table 2 |
| **Risk of bias in individual studies** | 14 | Describe anticipated methods for assessing risk of bias of individual studies, including whether this will be done at the outcome or study level, or both; state how this information will be used in data synthesis |  |  | Line 303-344 |
| ***DATA*** | | | | | |
| **Synthesis** | 15a | Describe criteria under which study data will be quantitatively synthesized |  |  | Line 371-376 |
|  | 15b | If data are appropriate for quantitative synthesis, describe planned summary measures, methods of handling data, and methods of combining data from studies, including any planned exploration of consistency (e.g., *I* ^2^, Kendall’s tau) |  |  | Line 378-385 |
|  | 15c | Describe any proposed additional analyses (e.g., sensitivity or subgroup analyses, meta-regression) |  |  | Line 385-418 |
|  | 15d | If quantitative synthesis is not appropriate, describe the type of summary planned |  |  |  |
| **Meta-bias(es)** | 16 | Specify any planned assessment of meta-bias(es) (e.g., publication bias across studies, selective reporting within studies) |  |  | Line 403-408 |
| **Confidence in cumulative evidence** | 17 | Describe how the strength of the body of evidence will be assessed (e.g., GRADE) |  |  | Line 345-369 |

**Supplement 2.** List of 30 benchmark papers used for search sensitivity evaluations

| **Nr** | **Reference** | **DOI** |
| --- | --- | --- |
| 1 | Anschutz DJ, Engels RC & Van Strien T (2009). Side effects of television food commercials on concurrent nonadvertised sweet snack food intakes in young children.. American Journal of Clinical Nutrition, 89(5), 1328-33. https://dx.doi.org/10.3945/ajcn.2008.27075 | 10.3945/ajcn.2008.27075 |
| 2 | Forman, J., Halford, J. C., Summe, H., MacDougall, M., & Keller, K. L. (2009). Food branding influences ad libitum intake differently in children depending on weight status. Results of a pilot study. Appetite, 53(1), 76–83. https://doi.org/10.1016/j.appet.2009.05.015 | 10.1016/j.appet.2009.05.015 |
| 3 | Harris, J. L., Bargh, J. A., & Brownell, K. D. (2009). Priming effects of television food advertising on eating behavior. Health psychology : official journal of the Division of Health Psychology, American Psychological Association, 28(4), 404–413. https://doi.org/10.1037/a0014399 | 10.1037/a0014399 |
| 4 | Anschutz, D. J., Engels, R. C., & Van Strien, T. (2010). Maternal encouragement to be thin moderates the effect of commercials on children's snack food intake. Appetite, 55(1), 117–123. https://doi.org/10.1016/j.appet.2010.03.014 | 10.1016/j.appet.2010.03.014 |
| 5 | Dovey, T. M., Taylor, L., Stow, R., Boyland, E. J., & Halford, J. C. (2011). Responsiveness to healthy television (TV) food advertisements/commercials is only evident in children under the age of seven with low food neophobia. Appetite, 56(2), 440–446. https://doi.org/10.1016/j.appet.2011.01.017 | 10.1016/j.appet.2011.01.017 |
| 6 | Harris, J. L., Speers, S. E., Schwartz, M. B., & Brownell, K. D. (2012). US food company branded advergames on the internet: children's exposure and effects on snack consumption. Journal of children and media, 6(1), 51-68. https://doi.org/10.1080/17482798.2011.633405 | 10.1080/17482798.2011.633405 |
| 7 | Keller, K. L., Kuilema, L. G., Lee, N., Yoon, J., Mascaro, B., Combes, A. L., Deutsch, B., Sorte, K., & Halford, J. C. (2012). The impact of food branding on children's eating behavior and obesity. Physiology & behavior, 106(3), 379–386. https://doi.org/10.1016/j.physbeh.2012.03.011 | 10.1016/j.physbeh.2012.03.011 |
| 8 | Kotler, J. A., Schiffman, J. M., & Hanson, K. G. (2012). The influence of media characters on children's food choices. Journal of health communication, 17(8), 886–898. https://doi.org/10.1080/10810730.2011.650822 | 10.1080/10810730.2011.650822 |
| 9 | Gregori, D., Ballali, S., Gafare, C. E., Casella, A., Stefanini, G., de Sousa Alves, R., Franchin, L., Amador, I., Da Silva, N. M., & Dibildox, J. (2013). Investigating the obesogenic effects of marketing snacks with toys: an experimental study in Latin America. Nutrition journal, 12, 95. https://doi.org/10.1186/1475-2891-12-95 | 10.1186/1475-2891-12-95 |
| 10 | Folkvord, F., Anschütz, D. J., Buijzen, M., & Valkenburg, P. M. (2013). The effect of playing advergames that promote energy-dense snacks or fruit on actual food intake among children. The American journal of clinical nutrition, 97(2), 239–245. https://doi.org/10.3945/ajcn.112.047126 | 10.3945/ajcn.112.047126 |
| 11 | Boyland, E. J., Harrold, J. A., Dovey, T. M., Allison, M., Dobson, S., Jacobs, M. C., & Halford, J. C. (2013). Food choice and overconsumption: effect of a premium sports celebrity endorser. Journal of Pediatrics, 163(2), 339–343. https://doi.org/10.1016/j.jpeds.2013.01.059 | 10.1016/j.jpeds.2013.01.059 |
| 12 | Gregori, D., Gulati, A., Hochdorn, A., Ballali, S., Paramesh, H., Kumar, M., & Baldi, I. (2014). Food packaged with toys: an investigation on potential obesogenic effects in Indian children. Indian journal of pediatrics, 81 Suppl 1, 30–38. https://doi.org/10.1007/s12098-014-1448-x | 10.1007/s12098-014-1448-x |
| 13 | Folkvord, F., Anschütz, D. J., Nederkoorn, C., Westerik, H., & Buijzen, M. (2014). Impulsivity, "advergames," and food intake. Pediatrics, 133(6), 1007–1012. https://doi.org/10.1542/peds.2013-3384 | 10.1542/peds.2013-3384 |
| 14 | Anderson, G. H., Khodabandeh, S., Patel, B., Luhovyy, B. L., Bellissimo, N., & Mollard, R. C. (2015). Mealtime exposure to food advertisements while watching television increases food intake in overweight and obese girls but has a paradoxical effect in boys. Applied physiology, nutrition, and metabolism = Physiologie appliquee, nutrition et metabolisme, 40(2), 162–167. https://doi.org/10.1139/apnm-2014-0249 | 10.1139/apnm-2014-0249 |
| 15 | Folkvord, F., Anschütz, D. J., Wiers, R. W., & Buijzen, M. (2015). The role of attentional bias in the effect of food advertising on actual food intake among children. Appetite, 84, 251–258. https://doi.org/10.1016/j.appet.2014.10.016 | 10.1016/j.appet.2014.10.016 |
| 16 | Kelly, B., Freeman, B., King, L., Chapman, K., Baur, L. A., & Gill, T. (2016). Television advertising, not viewing, is associated with negative dietary patterns in children. Pediatric obesity, 11(2), 158–160. https://doi.org/10.1111/ijpo.12057 | 10.1111/ijpo.12057 |
| 17 | Folkvord, F., Veling, H., & Hoeken, H. (2016). Targeting implicit approach reactions to snack food in children: Effects on intake. Health psychology : official journal of the Division of Health Psychology, American Psychological Association, 35(8), 919–922. https://doi.org/10.1037/hea0000365 | 10.1037/hea0000365 |
| 18 | Emond, J. A., Lansigan, R. K., Ramanujam, A., & Gilbert-Diamond, D. (2016). Randomized Exposure to Food Advertisements and Eating in the Absence of Hunger Among Preschoolers. Pediatrics, 138(6), e20162361. https://doi.org/10.1542/peds.2016-2361 | 10.1542/peds.2016-2361 |
| 19 | Gregori, D., Lorenzoni, G., Ballali, S., Vecchio, M. G., Verduci, E., Berchialla, P. (2017). Is brand visibility on snacks packages affecting their consumption in children? Results from an experimental ad-libitum study. Archivos Latinoamericanos de Nutricion, 67(1), 36-49. | Could not find by title or DOI – not indexed in the Medline database. |
| 20 | Folkvord, F., Lupiáñez-Villanueva, F., Codagnone, C., Bogliacino, F., Veltri, G., & Gaskell, G. (2017). Does a 'protective' message reduce the impact of an advergame promoting unhealthy foods to children? An experimental study in Spain and The Netherlands. Appetite, 112, 117–123. https://doi.org/10.1016/j.appet.2017.01.026 | 10.1016/j.appet.2017.01.026 |
| 21 | Gilbert-Diamond, D., Emond, J. A., Lansigan, R. K., Rapuano, K. M., Kelley, W. M., Heatherton, T. F., & Sargent, J. D. (2017). Television food advertisement exposure and FTO rs9939609 genotype in relation to excess consumption in children. International journal of obesity (2005), 41(1), 23–29. https://doi.org/10.1038/ijo.2016.163 | 10.1038/ijo.2016.163 |
| 22 | Harris, J. L., Haraghey, K. S., Lodolce, M., & Semenza, N. L. (2017). Teaching children about good health? Halo effects in child-directed advertisements for unhealthy food. Pediatric obesity, 13(4), 256–264. https://doi.org/10.1111/ijpo.12257 | 10.1111/ijpo.12257 |
| 23 | Brown, C. L., Matherne, C. E., Bulik, C. M., Howard, J. B., Ravanbakht, S. N., Skinner, A. C., Wood, C. T., Bardone-Cone, A. M., Brown, J. D., Perrin, A. J., Levine, C., Steiner, M. J., & Perrin, E. M. (2017). Influence of product placement in children's movies on children's snack choices. *Appetite*, 114, 118–124. https://doi.org/10.1016/j.appet.2017.03.022 | 10.1016/j.appet.2017.03.022 |
| 24 | Dalton, M. A., Longacre, M. R., Drake, K. M., Cleveland, L. P., Harris, J. L., Hendricks, K., & Titus, L. J. (2017). Child-targeted fast-food television advertising exposure is linked with fast-food intake among pre-school children. Public health nutrition, 20(9), 1548–1556. https://doi.org/10.1017/S1368980017000520 | 10.1017/S1368980017000520 |
| 25 | Aerts, G., & Smits, T. (2019). Do depicted suggestions of portion size on‐pack impact how much (un) healthy food children consume. International Journal of Consumer Studies, 43(3), 237-244. https://doi.org/10.1111/ijcs.12503 | Could not find by title or DOI – not indexed in the Medline database. |
| 26 | Fernandez, M., Februhartanty, J., & Bardosono, S. (2019). Association between food marketing exposure and consumption of confectioneries among pre-school children in Jakarta. Malaysian Journal of Nutrition, 63-73. | Could not find by title or DOI – not indexed in the Medline database. |
| 27 | Coates, A. E., Hardman, C. A., Halford, J. C. G., Christiansen, P., & Boyland, E. J. (2019). Social Media Influencer Marketing and Children's Food Intake: A Randomized Trial. Pediatrics, 143(4), e20182554. https://doi.org/10.1542/peds.2018-2554 | 10.1542/peds.2018-2554 |
| 28 | Coates, A. E., Hardman, C. A., Halford, J. C. G., Christiansen, P., & Boyland, E. J. (2019). The effect of influencer marketing of food and a “protective” advertising disclosure on children's food intake. *Pediatric obesity, 14*(10), e12540. https://doi.org/10.1111/ijpo.12540 | 10.1111/ijpo.12540 |
| 29 | Leonard, B., Campbell, M. C., & Manning, K. C. (2019). Kids, Caregivers, and Cartoons: The Impact of Licensed Characters on Food Choices and Consumption. Journal of Public Policy & Marketing, 38(2), 214–231. https://doi.org/10.1177/0743915619827919 | Could not find by title or DOI – not indexed in the Medline database. |
| 30 | Emond, J. A., Longacre, M. R., Drake, K. M., Titus, L. J., Hendricks, K., MacKenzie, T., Harris, J. L., Carroll, J. E., Cleveland, L. P., Langeloh, G., & Dalton, M. A. (2019). Exposure to Child-Directed TV Advertising and Preschoolers' Intake of Advertised Cereals. American journal of preventive medicine, 56(2), e35–e43. https://doi.org/10.1016/j.amepre.2018.09.015 | 10.1016/j.amepre.2018.09.015 |

**Supplement 3.** Literature search string sensitivity evaluation.

| **Date / database** | **Search nr** | **Search string** | **Search result / comment** |
| --- | --- | --- | --- |
| 2024 July 24 / Ovid MEDLINE(R) ALL <1946 to September 04, 2025> | 1 | *("10.3945/ajcn.2008.27075" or "10.1016/j.appet.2009.05.015" or "10.1037/a0014399" or "10.1016/j.appet.2010.03.014" or "10.1016/j.appet.2011.01.017" or "10.1080/17482798.2011.633405" or "10.1016/j.physbeh.2012.03.011" or "10.1080/10810730.2011.650822" or "10.1186/1475-2891-12-95" or "10.3945/ajcn.112.047126" or "10.1016/j.jpeds.2013.01.059" or "10.1007/s12098-014-1448-x" or "10.1542/peds.2013-3384" or "10.1139/apnm-2014-0249" or "10.1016/j.appet.2014.10.016" or "10.1111/ijpo.12057" or "10.1037/hea0000365" or "10.1542/peds.2016-2361" or "10.1016/j.appet.2017.01.026" or "10.1038/ijo.2016.163" or "10.1111/ijpo.12257" or "10.1016/j.appet.2017.03.022" or "10.1017/S1368980017000520" or "10.1111/ijcs.12503" or "10.1542/peds.2018-2554" or "10.1111/ijpo.12540" or "10.1177/0743915619827919" or "10.1016/j.amepre.2018.09.015").do.* | 25 /  25 out of 30 benchmark studies found, 5 benchmark studies absent from the database – could not find by DOI or title |
|  | 2 | *(((Beverages/ or Carbonated Beverages/ or Energy Drinks/ or exp Food/ or exp Food Industry/ or "fruit and vegetable juices"/ or exp milk/ or exp Milk Substitutes/ or sugar-sweetened beverages/ or exp Tea/ or Teas, Herbal/ or Diet/ or (beverage* or diet* or drink* or food* or nutrition* or snack*).ti,kw,kf.) and (Advertising as Topic/ or Direct-to-Consumer Advertising/ or exp Marketing/ or (adspend* or advert* or advergame* or commercial or commercials or market* or promot* or sponsor*).ti,ab,kw,kf.)) or ((beverage* or diet* or drink* or fast-food* or food* or nutrition* or snack*) adj3 (adspend* or advert* or advergame* or commercial or commercials or market* or promot* or sponsor*)).ab.) and (exp Adolescent/ or exp Child/ or exp Infant/ or exp Schools/ or (adolescen* or baby or babies or child* or infant* or pupil* or teen* or "young people" or "young person" or youth*).ti,ab,kw,kf.) and (exp Eating/ or Energy Intake/ or Feeding Behavior/ or Food Preferences/ or (calori* or choice* or consum* or eating behavio* or energ* or favor* or favour* or intake* or liking or prefer* or snacking behavio*).ti,ab,kw,kf.)* | 13434 / Target search string |
|  | 3 | *1 and 2* | 25 / Sensitivity is 25/25 = 1 (100%). |

**Supplement 4:** Search history

| **Ovid MEDLINE(R) ALL <1946 to September 04, 2025>**  1 Beverages/ or Carbonated Beverages/ or Diet/ or Energy Drinks/ or exp Food/ or exp Food Industry/ or "fruit and vegetable juices"/ or exp Milk/ or exp Milk Substitutes/ or sugar-sweetened beverages/ or exp Tea/ or Teas, Herbal/ 1753807  2 (beverage* or diet* or drink* or food* or nutrition* or snack*).ti,kw,kf. 661790  3 1 or 2 2091901  4 Advertising as Topic/ or Direct-to-Consumer Advertising/ or exp Marketing/ 38318  5 (adspend* or advert* or advergame* or commercial or commercials or market* or promot* or sponsor*).ti,ab,kw,kf. 1910876  6 4 or 5 1926607  7 ((beverage* or diet* or drink* or fast-food* or food* or nutrition* or snack*) adj3 (adspend* or advert* or advergame* or commercial or commercials or market* or promot* or sponsor*)).ab. 22504  8 (3 and 6) or 7 183889  9 exp Adolescent/ or exp Child/ or exp Child, preschool/ or exp Infant/ or exp Schools/ 4199914  10 (adolescen* or baby or babies or child* or infant* or pupil* or teen* or "young people" or "young person" or youth*).ti,ab,kw,kf. 2484554  11 9 or 10 4855524  12 exp Eating/ or Energy Intake/ or Feeding Behavior/ or Food Preferences/ 214060  13 (calori* or choice* or consum* or eating behavio* or energ* or favor* or favour* or intake* or liking or prefer* or snacking behavio*).ti,ab,kw,kf. 3246785  14 12 or 13 3332891  15 8 and 11 and 14 13434  16 limit 15 to ed=20200301-20241028 **3821** |
| --- |
| **Embase <1974 to 2025 September 03>**  1 Diet/ or exp Food/ 1519643  2 (beverage* or diet* or drink* or food* or nutrition* or snack*).ti,kw,kf. 767288  3 1 or 2 1943459  4 exp Advertising/ or Marketing/ 38110  5 (adspend* or advert* or advergame* or commercial or commercials or market* or promot* or sponsor*).ti,ab,kw,kf. 2359362  6 4 or 5 2359362  7 ((beverage* or diet* or drink* or fast-food* or food* or nutrition* or snack*) adj3 (adspend* or advert* or advergame* or commercial or commercials or market* or promot* or sponsor*)).ab. 26898  8 (3 and 6) or 7 211439  9 exp Adolescent/ or exp Child/ or exp Schools/ 4487883  10 (adolescen* or baby or babies or child* or infant* or pupil* or teen* or "young people" or "young person" or youth*).ti,ab,kw,kf. 3068080  11 9 or 10 5270038  12 exp Eating/ or exp Dietary Intake/ or Feeding Behavior/ or Food Preference/ 804268  13 (calori* or choice* or consum* or eating behavio* or energ* or favor* or favour* or intake* or liking or prefer* or snacking behavio*).ti,ab,kw,kf. 3936222  14 12 or 13 4333380  15 8 and 11 and 14 18144  16 limit 15 to dd=20200301-20241028 **1827** |
| **EBM Reviews - Cochrane Database of Systematic Reviews <2005 to August 27, 2025>**  **EBM Reviews - Cochrane Central Register of Controlled Trials <August 2025>**  1 ((beverage* or diet* or drink* or food* or juice* or milk* or nutrition* or snack* or tea*) adj3 (adspend* or advert* or advergame* or commercial or commercials or market* or promot* or sponsor*)).ti,ab,kw,kf. 3346  2 (adolescen* or baby or babies or child* or infant* or pupil* or teen* or "young people" or "young person" or youth*).ti,ab,kw,kf. 267294  3 (calori* or choice* or consum* or eating behavio* or energ* or favor* or favour* or intake* or liking or prefer* or snacking behavio*).ti,ab,kw,kf. 300442  4 1 and 2 and 3 650  5 limit 4 to yr="2020-2024" **210** |
| **Web of Science^1^**  #1 TS=( (beverage* OR diet* OR drink* OR food* OR juice* OR milk* OR nutrition* OR snack* OR tea*) NEAR/3 (adspend* OR advert* OR advergame* OR commercial OR commercials OR market* OR promot* OR sponsor*) ) 76164  #2 TS=(adolescen* OR baby OR babies OR child* OR infant* OR pupil* OR teen* OR "young people" OR "young person" OR youth* OR schools) 3784830  #3 TS=(calori* OR choice* OR consum* OR "eating behavior" OR "feeding behavior" OR "food preferences" OR intake* OR liking OR prefer* OR "snacking behavior" OR "energy intake") 6812482  #4 #1 AND #2 AND #3 5918  #5 #4 Timespan: 2020-03-01 to 2024-10-31 [Publication Date] **2228** |
| **CINAHL Complete^1^**  S1 (MH "Beverages") or (MH "Carbonated Beverages") or (MH "Energy Drinks") or (MH "Fruit Juices+") or (MH "Food+") or (MH "Kombucha") or (MH "Milk") or (MH "Milk Substitutes+") or (MH "Sweetened Beverages") or (MH "Tea+") or (MH "Food Industry+") or (MH "Diet") OR (TI(beverage* or diet* or drink* or food* or nutrition* or snack*)) 378908  S2 (MH "Marketing+") or ((TI(adspend* or advert* or advergame* or commercial or commercials or market* or promot* or sponsor*)) or (AB(adspend* or advert* or advergame* or commercial or commercials or market* or promot* or sponsor*)) 345518  S3 (AB((beverage* or diet* or drink* or fast-food* or food* or nutrition* or snack*) N3 (adspend* or advert* or advergame* or commercial or commercials or market* or promot* or sponsor*))) 7737  S4 S1 AND S2 29860  S5 S3 OR S4 31395  S6 (MH "Adolescence+") or (MH "Child+") or (MH "Schools+") or ((TI(adolescen* or baby or babies or child* or infant* or pupil* or teen* or "young people" or "young person" or youth*)) or (AB(adolescen* or baby or babies or child* or infant* or pupil* or teen* or "young people" or "young person" or youth*))) 1474132  S7 (MH "Eating") or (MH "Eating Behavior") or (MH "Energy Intake") or (MH "Food Intake+") or (MH "Food Preferences") or (AB(calori* or choice* or consum* or eating behavio* or energ* or favor* or favour* or intake* or liking or prefer* or snacking behavio*)) or (TI(calori* or choice* or consum* or eating behavio* or energ* or favor* or favour* or intake* or liking or prefer* or snacking behavio*)) 570537  S8 S5 AND S6 AND S7 6235  S9 S8 Publication date: 20200301-20241031 **1864** |
| **Business Source Ultimate**  S1 (DE "BEVERAGE industry") OR (DE "FOOD industry") 93950  S2 (TI(beverage* or diet* or drink* or food* or nutrition* or snack*)) 165964  S3 S1 OR S2 235489  S4 (DE "ADVERTISING & children") OR (DE "INTERNET advertising & children") OR (DE “ADVERTISING & minorities”) OR (DE "MARKETING") 220147  S5 (TI(adspend* or advert* or advergame* or commercial or commercials or market* or promot* or sponsor*)) or (AB(adspend* or advert* or advergame* or commercial or commercials or market* or promot* or sponsor*)) or (KW(adspend* or advert* or advergame* or commercial or commercials or market* or promot* or sponsor*)) 3986145  S6 S4 OR S5 4026691  S7 (AB((beverage* or diet* or drink* or fast-food* or food* or nutrition* or snack*) N3 (adspend* or advert* or advergame* or commercial or commercials or market* or promot* or sponsor*))) 45142  S8 (S3 AND S6) OR S7 89233  S9 (DE "CHILD consumers") OR (DE "STUDENTS as consumers") OR (DE "TEENAGE consumers") OR (DE "YOUNG consumers") OR (DE “MINORITY consumers”) 6600  S10 (TI(adolescen* or baby or babies or child* or infant* or pupil* or teen* or "young people" or "young person" or youth*)) or (AB(adolescen* or baby or babies or child* or infant* or pupil* or teen* or "young people" or "young person" or youth*)) or (KW(adolescen* or baby or babies or child* or infant* or pupil* or teen* or "young people" or "young person" or youth*)) 406712  S11 S9 OR S10 410107  S12 (DE "BEVERAGE consumption statistics") OR (DE "FOOD consumption statistics") 27  S13 (AB(calori* or choice* or consum* or eating behavio* or energ* or favor* or favour* or intake* or liking or prefer* or snacking behavio*)) OR (TI(calori* or choice* or consum* or eating behavio* or energ* or favor* or favour* or intake* or liking or prefer* or snacking behavio*)) OR (KW(calori* or choice* or consum* or eating behavio* or energ* or favor* or favour* or intake* or liking or prefer* or snacking behavio*)) 1489  S14 S12 OR S13 1952734  S15 S8 AND S11 AND S14 1489  S16 S15 Publication date: 20200301-20241031 **321** |
| **Communication & Mass Media Complete^1^**  S1 (TI(beverage* or diet* or drink* or food* or juice* or milk* or nutrition* or snack* or tea*)) 22430  S2 (DE "ADVERTISING & children") OR (DE "INTERNET advertising & children") OR (DE “ADVERTISING & minorities”) OR (DE "MARKETING") 21466  S3 (TI(adspend* or advert* or advergame* or commercial or commercials or market* or promot* or sponsor*)) or (AB(adspend* or advert* or advergame* or commercial or commercials or market* or promot* or sponsor*)) or (KW(adspend* or advert* or advergame* or commercial or commercials or market* or promot* or sponsor*)) 180261  S4 S1 AND (S2 OR S3) 3544  S5 (DE "JUNK food advertising") OR (DE "FAST food restaurant advertising") 26  S6 (AB((beverage* or diet* or drink* or fast-food* or food* or nutrition* or snack*) N3 (adspend* or advert* or advergame* or commercial or commercials or market* or promot* or sponsor*)) ) 2608  S7 S4 OR S5 OR S6 5317  S8 (TI(adolescen* or baby or babies or child* or infant* or pupil* or teen* or "young people" or "young person" or youth*)) or (AB(adolescen* or baby or babies or child* or infant* or pupil* or teen* or "young people" or "young person" or youth*)) or (KW(adolescen* or baby or babies or child* or infant* or pupil* or teen* or "young people" or "young person" or youth*)) 62124  S9 (AB(calori* or choice* or consum* or eating behavio* or energ* or favor* or favour* or intake* or liking or prefer* or snacking behavio*) OR (TI(calori* or choice* or consum* or eating behavio* or energ* or favor* or favour* or intake* or liking or prefer* or snacking behavio*)) OR (KW(calori* or choice* or consum* or eating behavio* or energ* or favor* or favour* or intake* or liking or prefer* or snacking behavio*)) 95234  S10 S7 AND S8 AND S9 261  S11 S10 Publication date: 20200301-20241031 **31** |
| **ERIC^1^**  S1 (DE “FOOD”) 6651  S2 (TI(beverage* or diet* or drink* or food* or nutrition* or snack*)) 8374  S3 S1 OR S2 12218  S4 (DE "ADVERTISING") OR (DE "MARKETING") 11501  S5 (TI(adspend* or advert* or advergame* or commercial or commercials or market* or promot* or sponsor*)) or (AB(adspend* or advert* or advergame* or commercial or commercials or market* or promot* or sponsor*)) 171089  S6 S4 OR S5 172942  S7 (AB((beverage* or diet* or drink* or fast-food* or food* or nutrition* or snack*) N3 (adspend* or advert* or advergame* or commercial or commercials or market* or promot* or sponsor*)) ) 929  S8 (S3 AND S6) OR S7 2329  S9 (DE "Young Children") OR (DE "Infants") OR (DE "Preschool Children") OR (DE "Toddlers") OR (DE "Children") OR (DE "Youth") OR (DE "Adolescents") OR (DE "Early Adolescents") OR (DE "Late Adolescents") 173341  S10 (TI(adolescen* or baby or babies or child* or infant* or pupil* or teen* or "young people" or "young person" or youth*)) or (AB(adolescen* or baby or babies or child* or infant* or pupil* or teen* or "young people" or "young person" or youth*)) 445654  S11 S9 OR S10 462989  S12 (DE "Eating Habits") 4391  S13 (AB(calori* or choice* or consum* or eating behavio* or energ* or favor* or favour* or intake* or liking or prefer* or snacking behavio*)) OR (TI(calori* or choice* or consum* or eating behavio* or energ* or favor* or favour* or intake* or liking or prefer* or snacking behavio*)) 157952  S14 S12 OR S13 160213  S15 S8 AND S11 AND S14 517  S16 S15 Publication date: 20200301-20241031 **62** |
| **Index to Legal Periodicals and Books (H.W. Wilson) ^1^**  S1 (AB((beverage* OR diet* OR drink* OR food* OR juice* OR milk* OR nutrition* OR snack* OR tea*) N3 (adspend* or advert* or advergame* or commercial or commercials or market* or promot* or sponsor*))) OR (TI((beverage* OR diet* OR drink* OR food* OR juice* OR milk* OR nutrition* OR snack* OR tea*) N3 (adspend* or advert* or advergame* or commercial or commercials or market* or promot* or sponsor*))) 703  S2 (DE "Teenagers") OR (DE "Students") 729  S3 (TI(adolescen* or baby or babies or child* or infant* or pupil* or teen* or "young people" or "young person" or youth*)) or (AB(adolescen* or baby or babies or child* or infant* or pupil* or teen* or "young people" or "young person" or youth*)) 28244  S4 S2 OR S3 28763  S5 (AB(calori* or choice* or consum* or eating behavio* or energ* or favor* or favour* or intake* or liking or prefer* or snacking behavio*)) OR (TI(calori* or choice* or consum* or eating behavio* or energ* or favor* or favour* or intake* or liking or prefer* or snacking behavio*)) 42629  S6 S1 AND S4 AND S5 34  S7 S6 Publication date: 20200301-20241031 **6** |
| **EconLit**  S1 MAINSUBJECT.EXACT("Food; Beverages; Cosmetics; Tobacco; Wine and Spirits (L66)") 14370  S2 title(beverage* OR diet* OR drink* OR food* OR nutrition* OR snack*) 18422  S3 [S1] OR [S2] 28125  S4 MAINSUBJECT.EXACT("Marketing and Advertising (M3)") OR MAINSUBJECT.EXACT("Advertising (M37)") OR MAINSUBJECT.EXACT("Marketing (M31)") OR MAINSUBJECT.EXACT("Marketing and Advertising: General (M30)") 25111  S5 abstract(adspend* OR advert* OR advergame* OR commercial OR commercials OR market* OR promot* OR sponsor*) OR title(adspend* OR advert* OR advergame* OR commercial OR commercials OR market* OR promot* OR sponsor*) 406463  S6 [S4] OR [S5] 415576  S7 abstract((beverage* OR diet* OR drink* OR fast-food* OR food* OR nutrition* OR snack*) NEAR/3 (adspend* OR advert* OR advergame* OR commercial OR commercials OR market* OR promot* OR sponsor*)) 2159  S8 ([S3] AND [S6]) OR [S7] 10035  S9 title(adolescen* or baby or babies or child* or infant* or pupil* or teen* or "young people" or "young person" or youth*) or abstract(adolescen* or baby or babies or child* or infant* or pupil* or teen* or "young people" or "young person" or youth*) 47071  S10 title(calori* or choice* or consum* or eating behavio* or energ* or favor* or favour* or intake* or liking or prefer* or snacking behavio*) OR abstract(calori* or choice* or consum* or eating behavio* or energ* or favor* or favour* or intake* or liking or prefer* or snacking behavio*) 284635  S11 [S8] AND [S9] AND [S10] 264  S12 [S11] AND pd(20200301-20241031) **66** |
| **Emerald^1^**  (beverage* OR diet* OR drink* OR fast-food* OR food* OR nutrition* OR snack*)  AND  (adspend* OR advert* OR advergame* OR commercial OR commercials OR market* OR promot* OR sponsor*)  AND  (adolescen* OR baby OR babies OR child* OR infant* OR pupil* OR teen* OR "young people" OR "young person" OR youth* OR schools)  AND  (calori* or choice* or consum* or eating behavio* or energ* or favor* or favour* or intake* or liking or prefer* or snacking behavio*)  AND  Data range: 2020-2024 **0** |
| **Database of Promoting Health Effectiveness Reviews (DoPHER)**  1 Freetext (All but Author): ("beverage*" or "diet*" or "drink*" or "food*" or "juice*" or "milk*" or "nutrition*" or "snack*" or "tea*") 2441  2 Freetext (All but Author): ("adspend*" or "advert*" or "advergame*" or commerical or commericals or "market*" or "promot*" or "sponsor*") 2701  3 Freetext (All but Author): ("adolescen*" or baby or babies or "child*" or "infant*" or "pupil*" or "teen*" or "young people" or "young person" or "youth*" or schools) 3536  4 Freetext (All but Author): ("calori*" or "choice*" or "consum*" or "eating behavio*" or "energ*" or "favor*" or "favour*" or "intake*" or liking or "prefer*" or "snacking behavio*") 2075  6 Freetext (Year): >2020 2542  7 1 AND 2 AND 3 AND 4 AND 5 **56** |
| **Global Index Medicus^1^**  (MH: (G07.203.300* or J02.500*) OR TW: (beverage* or diet* or drink* or food* or nutrition* or snack*)) AND (MH: (“Marketing” or J01.219.687.274*) OR TW: (adspend* or advert* or advergame* or commercial or commercials or market* or promot* or sponsor*)) AND  (MH: (“Adolescent” or M01.060.406* or M01.060.703*) OR TW: (adolescen* or baby or babies or child* or infant* or pupil* or teen* or "young people" or "young person" or youth*)) AND  (MH: (“Feeding Behavior” or “Energy Intake” or “Food Preferences”) OR TW: (calori* or choice* or consum* or eating behavio* or energ* or favor* or favour* or intake* or liking or prefer* or snacking behavio*))  AND  (DA: (202003 or 202004 or 202005 or 202006 or 202007 or 202008 or 202009 or 202010 or 202011 or 202012 or 2021$ or 2022$ or 2023$ or 2024$)) **245** |
| **Health Evidence^1^**  (beverage* or diet* or drink* or food* or juice* or milk* or nutrition* or snack* or tea*)  AND  (adspend* or advert* or advergame* or commerical or commericals or market* or promot* or sponsor*)  AND  (adolescen* or baby or babies or child* or infant* or pupil* or teen* or young people or young person or youth* or schools )  AND  (calori* or choice* or consum* or eating behavio* or energ* or favor* or favour* or intake* or liking or prefer* or snacking behavio* )  AND  Date = Published from 2020 to 2024 **127** |
| **Institutional Repository of Information Sharing (IRIS) ^1^**  (dc.subject.mesh:"beverages” or dc.subject.mesh:"food” or dc.title:(beverage* OR diet* OR drink* OR food* OR juice* OR milk* OR nutrition* OR snack* OR tea*) or dc.description.abstract:(beverage* OR diet* OR drink* OR food* OR juice* OR milk* OR nutrition* OR snack* OR tea*) )  AND  (dc.subject.mesh:"Advertising” or dc.subject.mesh:"marketing” or dc.title:( adspend* or advert* or advergame* or commerical or commericals or market* or promot* or sponsor*) or dc.description.abstract:( adspend* or advert* or advergame* or commerical or commericals or market* or promot* or sponsor*) )  AND  (dc.subject.mesh:"child” or dc.subject.mesh:"infant” or dc.subject.mesh:"adolescent” or dc.title:(adolescen* or baby or babies or child* or infant* or pupil* or teen* or young people or young person or youth* or schools) or dc.description.abstract:( adolescen* or baby or babies or child* or infant* or pupil* or teen* or young people or young person or youth* or schools))  AND  (dc.subject.mesh:"eating” or dc.title:( calori* or choice* or consum* or eating behavio* or energ* or favor* or favour* or intake* or liking or prefer* or snacking behavio*) or dc.description.abstract:( calori* or choice* or consum* or eating behavio* or energ* or favor* or favour* or intake* or liking or prefer* or snacking behavio*) )  AND  (Date issued: [2020-3 TO 2024]) **100** |
| **JSTOR^1^**  (food* or beverage* or drink* or snack*)  AND  (advert* or market* or promot*)  AND  (adolescen* or child* or infant*)  AND  (Publication date: 2020/03/01 TO 2024/10/31) **3** |
| **KoreaMed^1^**  (food*[TIAB] OR beverage*[TIAB]) OR snack*[TIAB]) OR drink*[TIAB])  AND  (advert*[TIAB] OR market*[TIAB]) OR promot*[TIAB])  AND  (adolescen*[TIAB] OR child*[TIAB]) OR infant*[TIAB])  AND  (2020:2024[DPY]) **32** |
| **TRIP^1^**  beverage OR diet OR drink OR food OR juice OR milk OR nutrition OR snack OR tea,  adspend OR advert OR advergame OR commercial OR commercials OR market OR promot OR sponsor,  AND  adolescen OR baby OR babies OR child OR infant OR pupil OR teen OR "young people" OR "young person" OR youth OR schools,  AND  calori OR choice OR consum OR "eating behavior" OR "feeding behavior" OR "food preferences" OR intake OR liking OR prefer OR "snacking behavior" OR "energy intake"  AND  from_date:2020 to_date:2024 **17** |
| **Campbell Systematic Reviews^1^**  (“beverage* OR diet* OR drink* OR food* OR juice* OR milk* OR nutrition* OR snack* OR tea*”) anywhere  AND  (“adspend* OR advert* OR advergame* OR commercial OR commercials OR market* OR promot* OR sponsor*”) anywhere  AND  (“adolescen* OR baby OR babies OR child* OR infant* OR pupil* OR teen* OR "young people" OR "young person" OR youth* OR schools”) anywhere  AND  (“calori* OR choice* OR consum* OR "eating behavior" OR "feeding behavior" OR "food preferences" OR intake* OR liking OR prefer* OR "snacking behavior" OR "energy intake"”) anywhere  AND  (Publication date: 2020/03/01 TO 2024/10/31) **239** |

Footnote: ^1^ To ensure consistency across all 19 databases, search strings from databases that only allow a single block of terms were reformatted and presented by PICO concepts, with the total number of results shown in bold at the end of each cell. For databases that do not provide time-limit functions, publication date restrictions were also reformatted accordingly.

**Supplement 5.** Adopted Newcastle-Ottawa Quality Assessment Scale (NOS) for Cross-Sectional Studies

Customized for the systematic review protocol: "What is the effect of commercial food and non-alcoholic beverage marketing on the dietary intake of children and adolescents?" (PROSPERO CRD42025641870) based on NOS for cross-sectional studies in the Review by Blanchard et al: The effectiveness, cost-effectiveness and policy processes of regulatory, voluntary and partnership policies to improve food environments: an evidence synthesis. *National Institute for Health and Care Research* 2024.

**Selection**

1. Representativeness of the sample:
   1. Truly representative of the average in the target population (e.g., national surveys, random sampling). *(high quality)*
   2. Somewhat representative of the most common units in the target population (e.g., non-random sampling). *(moderate quality)*
   3. Selected group of users/convenience sample. *(low quality)*
   4. No description of the derivation of the included subjects. *(no info/unclear)*
2. Sample size:
   1. Justified in a satisfactory manner (e.g., sample size calculation provided or large sample clearly adequate [e.g., >1000]). *(high quality)*
   2. No formal sample size calculation, but sample is relatively large and precision is acceptable. (e.g., confidence intervals for the primary outcome are not wide, small standard errors). *(moderate quality)*
   3. Sample appears underpowered or non-representative *(low quality)*
   4. Not information provided. *(no info/unclear)*
3. Missing data:
   1. Complete data on exposure, outcome, and confounders are available for nearly all participants, or the analysis is based on a complete case analysis model that includes all or most predictors of missingness. *(high quality)*
   2. Some data on exposure, outcome, and/or confounder are missing, but there is evidence suggesting unbiased results (e.g., appropriate method used to correct bias, as shown by sensitivity analyses). *(moderate quality)*
   3. Evidence suggests bias due to missing data. *(low quality)*
   4. No information provided. *(no info/unclear)*
4. Ascertainment of exposure
   1. Exposure is well-characterized and accurately measured (e.g., validated tools used; the duration, intensity, and content of the measurement are aligned with the exposure of interest), or the measurement error presented is unlikely to bias the estimated effect of exposure on outcome (e.g., random non-differential measurement error). *(high quality)*
   2. Exposure is somewhat well-characterized and accurately measured (e.g., proxy report with limited validation, including the duration, intensity, or content of the measurement), or the measurement error presented is unlikely to bias the estimated effect of exposure on outcome *(moderate quality)*
   3. Exposure measures does not, or only minimally, characterize exposure of interest (e.g., perception-based measurement). *(low quality)*
   4. There is error or misclassification in the exposure measurement that is likely to bias the estimated effect of exposure on outcome. *(low quality)*
   5. No information provided on how exposure is measured. *(no info/unclear)*

**Comparability**

1. Comparability of subjects in different outcome groups on the basis of design or analysis. Confounding factors controlled.
   1. Data/results adjusted for all the important predictors/risk factors/confounders (i.e., age, SEP). *(high quality)*
   2. Data/results not adjusted for all important confounders/risk factors/information, but negative control or other consideration was used to justify there is no serious uncontrolled confounding. *(moderate quality)*
   3. Data/results not adjusted for at least one important predictors/risk factors/ confounder that is likely to have a material impact on the estimated effect of exposure on outcome. *(low quality)*

**Outcome**

1. Assessment of outcome:
   1. Food intake assessed using validated dietary assessment tools (e.g., 24-hour recall, FFQ, food diaries). *(high quality)*
   2. Self- or proxy-reported intake with some description but without validation *(moderate quality)*
   3. Poorly described or non-validated measures. *(low quality)*
   4. No information provided on how outcome is measured. *(no info/unclear)*
2. Statistical test
   1. Statistical test used to analyze the data clearly described, appropriate, and measures of association presented including confidence intervals or probability level (p-values). *(high quality)*
   2. Statistical test described is not optimal *(moderate quality)*
   3. Statistical test described is not appropriate *(low quality)*
   4. Statistical test not described or incomplete. *(no info/unclear)*

**Overall rating**

- Two key items: 4. Ascertainment of exposure and 7. Statistical test

**High quality:**

- Items 4 or 7 rated high, few other items rated moderate, no item rated low

**Moderate quality:**

- Items 4 or 7 rated moderate, and none rated low
- Judgement based on the other item ratings

**Low quality:**

- When 1+ item is rated low

**Unclear quality:**

- Items 4 or 7 rated unclear, and none rated low
- 2 + items rated unclear, and none rated low

**Supplement 6.** Adopted Newcastle-Ottawa Quality Assessment Scale (NOS) for Cohort Studies

Customized for the systematic review protocol: "What is the effect of commercial food and non-alcoholic beverage marketing on the dietary intake of children and adolescents?" (PROSPERO CRD42025641870) based on NOS for cohort studies in the Review by Geuder et al: Effects of Food Advertising on Youth’s Eating Behavior: A Systematic Review of Longitudinal Studies. *Clínica y Salud. Investigación Empírica en Psicología* 2025 36:2

Note: A study can be awarded a maximum of one point for each numbered item within the Selection and Outcome categories. A maximum of two points can be given for Comparability

**Selection**

1. Representativeness of the exposed cohort
   1. truly representative of the average children and adolescents (describe) in the community (*1 point*)
   2. somewhat representative of the average children and adolescents in the community (*1 point*)
   3. selected group of users eg nurses, volunteers
   4. no description of the derivation of the cohort
2. Selection of the non-exposed cohort
   1. drawn from the same community as the exposed cohort (*1 point*)
   2. drawn from a different source
   3. no description of the derivation of the non exposed cohort
3. Ascertainment of exposure
   1. secure record (eg surgical records) (*1 point*)
   2. structured interview (*1 point*)
   3. written self report
   4. no description

**Comparability**

1. Comparability of cohorts on the basis of the design or analysis
2. study controls for age and SEP (select the most important factor) (*1 point*)
3. study controls for any additional factor (e.g., sex, weight status, screen time, parental behaviour) (*1 point*)

**Outcome**

1. Assessment of outcome
2. independent blind assessment (*1 point*)
3. record linkage (*1 point*)
   1. self report
   2. no description
4. Was follow-up long enough for outcomes to occur
   1. yes (select an adequate follow up period for outcome of interest) *(1 point)*
   2. no
5. Adequacy of follow up of cohorts
6. complete follow up - all subjects accounted for (*1 point*)
7. subjects lost to follow up unlikely to introduce bias - small number lost - > 80 % (select an adequate %) follow up, or description provided of those lost) (*1 point*)
8. follow up rate < 80% (select an adequate %) and no description of those lost
9. no statement
